# Next-Generation Sequencing in the Field of Primary Immunodeficiencies: Current Yield, Challenges, and Future Perspectives

**DOI:** 10.1007/s12016-021-08838-5

**Published:** 2021-03-05

**Authors:** Emil E. Vorsteveld, Alexander Hoischen, Caspar I. van der Made

**Affiliations:** 1 Department of Human Genetics, Radboud University Medical Center, P.O. Box 9101, 6500 HB Nijmegen, The Netherlands; 2 Department of Internal Medicine, Radboudumc Center for Infectious Diseases (RCI), Radboudumc, Nijmegen, The Netherlands; 3 Radboud Institute for Molecular Life Sciences, Radboud University Medical Center, Nijmegen, The Netherlands

**Keywords:** Primary immunodeficiencies, Whole exome sequencing, Next-generation sequencing, Diagnostic yield, Precision medicine

## Abstract

Primary immunodeficiencies comprise a group of inborn errors of immunity that display significant clinical and genetic heterogeneity. Next-generation sequencing techniques and predominantly whole exome sequencing have revolutionized the understanding of the genetic and molecular basis of genetic diseases, thereby also leading to a sharp increase in the discovery of new genes associated with primary immunodeficiencies. In this review, we discuss the current diagnostic yield of this generic diagnostic approach by evaluating the studies that have employed next-generation sequencing techniques in cohorts of patients with primary immunodeficiencies. The average diagnostic yield for primary immunodeficiencies is determined to be 29% (range 10–79%) and 38% specifically for whole-exome sequencing (range 15–70%). The significant variation between studies is mainly the result of differences in clinical characteristics of the studied cohorts but is also influenced by varying sequencing approaches and (*in silico*) gene panel selection. We further discuss other factors contributing to the relatively low yield, including the inherent limitations of whole-exome sequencing, challenges in the interpretation of novel candidate genetic variants, and promises of exploring the non-coding part of the genome. We propose strategies to improve the diagnostic yield leading the way towards expanded personalized treatment in PIDs.

## Introduction

Primary immunodeficiencies (PIDs) are a group of inborn errors of immunity, caused by germline mutations that affect different parts of the immune system. PIDs are associated with a broad range of symptoms, including recurrent infections, autoimmunity, autoinflammation, allergies, and malignancy [1]. The diagnosis of these disorders is both hampered by the heterogeneous clinical presentation, genetic heterogeneity, and the variable mutational mechanisms that underlie the genetic defects. The advent of next-generation sequencing (NGS) has revolutionized the field of sequencing technologies, enabling high throughput sequencing with continuously decreasing costs and wide-spread use in both clinical and research settings. The application of these generic and unbiased techniques have also enabled an exponential increase in the identification of novel genes for PIDs, predominantly through the application of whole exome sequencing (WES) [2–4]. WES entails the sequencing of all protein coding exons and is widely used for the diagnosis of inherited disorders, enabling a genetic diagnosis that also provides insight into the molecular defect in PID patients, ultimately informing on the therapeutic options [5–7].

In this review, we discuss the application of WES in the context of PIDs and determine the current diagnostic yield from documented studies. In addition, we discuss the value of WES in research setting together with the limitations and pitfalls that are relevant for its use in diagnostics and research. Lastly, we discuss future perspectives, including the possible added value of whole genome sequencing (WGS) in the field of PIDs.

## Box 1 General Recommendations for the Analysis of NGS Data for PIDs

## WES is Superior to Targeted Sequencing as a First-Tier Diagnostic Approach

- WES facilitates analysis of (almost) all protein coding regions of the genome instead of a selected gene panel.
- WES *in silico* gene panels can be more easily updated than targeted sequencing panels as these leverage the existing exome data, while actual sequencing panels require a more laborious procedure and acquisition of new data.
- The diagnostic yield for PIDs using next-generation sequencing was on average 29%, and 38% for WES alone.
- Systematic and regular re-analysis of WES data and analysis of the whole exome in research setting improves yield [2, 8, 9].

## Analysis of WES Data: Prioritization and Strategies

- Variants called from WES data are subjected to standardized variant filtering for non-synonymous, rare variants impacting exons and splice sites.
- *In silico* gene lists are useful to find variants in described PID genes [1, 3, 10, 11]. When no suitable variant is found, all the genes outside of the PID genes can be analyzed to find potentially novel variants.
- To prioritize variants exome-wide, a detailed description of the patient phenotype and pedigree is helpful. The phenotype-genotype analysis requires judgement both at the variant and gene level. Metrics for the variant level include variant effect predictions, nucleotide alteration, and amino acid conservation. For the gene level, these include constraint against loss-of-function, functional/pathway annotations, and phenotypes of animal models such as knockout mice [12, 13].
- In sporadic cases of PIDs, a trio analysis can be performed by sequencing of both the patient and parents, allowing the exploration of *de novo* variants [14, 15]. In familial PID cases, variants from affected and/or unaffected family members can be overlapped to find candidate variants [3]. These approaches can drastically decrease the number of possible candidate variants.
- Functional testing in PID patients is minimally invasive and often required to demonstrate a possible functional defect for variants of uncertain significance (VUS).

## Opportunities to improve the Genetic Diagnosis of PID Patients

- There remains a high potential to find new genes associated with PIDs, also indicated by the low average yield seen for NGS-based methods in PIDs.
- Challenges in the diagnosis of PIDs include extremely rare or heterogeneous phenotypes, complex mutational mechanisms, or incomplete penetrance, which is in part due to the requirement of exposure to a specific pathogen to cause an overt phenotype.
- Collaborative efforts are being undertaken to match patients with similar genetic defects, especially in extremely rare PIDs, for example, by using genetic matchmaking’ platforms [2, 5, 16, 17].
- WGS starts to be used as a method to explore non-coding variation and its relevance in PIDs and has recently demonstrated its potential to diagnose patients with non-coding mutations [18].

## Genetics of PIDs

**Fig. 1:**
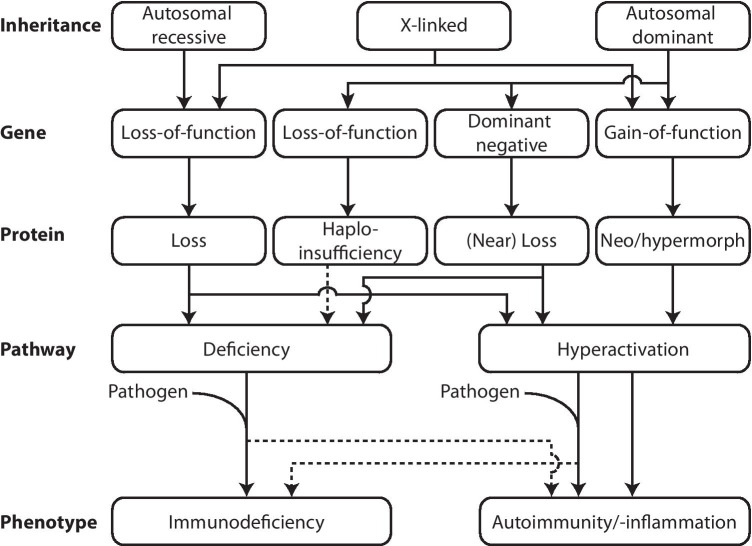
Schematic overview of the mutational mechanisms and effects on gene, protein, and pathway level with the phenotypic manifestations that result from various forms of PID. For autosomal recessive, X-linked and autosomal dominant forms of PID, the effects on the gene level and subsequently on the protein level are indicated that lead to either deficiency or hyperactivation of the immunological pathways involved. These effects at the pathway level can either result in a phenotype manifesting with symptoms of immunodeficiency, autoimmunity, or autoinflammation. Pathogen exposure can be a prerequisite for the phenotype to develop, which contributes to the incomplete penetrance observed in PIDs.

The number of PIDs and genes associated with inborn defects of immunity has sharply increased in recent years. Currently, over 400 different disorders have been described, arising from defects in 430 genes. Of all known PID genes, NGS already accounts for 45% of new gene discoveries [1]. The potential of NGS is reflected by the increasing number of recent discoveries, as 64 of the 430 known PID genes were discovered in the last 2 years [1]. The mutations known to cause PIDs influence all components of the intricate immune system and follow different inheritance patterns. Most of the established PIDs are inherited in an autosomal recessive fashion, caused by homozygous or compound heterozygous mutations, leading to a loss of function (LoF) of the encoded protein. In addition, heterozygous mutations inherited in an autosomal dominant (AD) fashion can exert LoF effects through haploinsufficiency or negative dominance, by causing an insufficient level of functional protein and through interference of the mutant protein with the wildtype protein, respectively. Lastly, in recent years, a growing number of heterozygous mutations have been identified that confer a hypermorphic or even neomorphic gain of function (GoF) effect, leading to an augmented or novel protein function [19]. The wide variety of genetic causes and the phenotypic heterogeneity of PID patients that pose an important diagnostic challenge are summarized in Fig. 1, where the relationship between mutational mechanism and effects on the gene, protein, and pathway is indicated, as well as the effect of the presence or absence of a pathogen on the clinical manifestations of PID patients.

Advances in the identification of new PID genes and the understanding of underlying disease mechanisms have revealed that different inheritance modes of mutations in the same gene exhibit different phenotypes. Illustratively, in the most recent classification of the International Union of Immunological Societies (IUIS), more than 35 genes are listed twice [1]. Examples of genes displaying such allelic series include *CARD11*, consisting of heterozygous mutations that cause distinct phenotypes through negative dominance (hyper IgE syndrome) or hypermorphism (B cell expansion with NF-kB and T cell anergy), whereas biallelic mutations lead to LoF ((severe) combined immunodeficiency) [20]. Moreover, both hypermorphic and LoF mutations in *STAT1* [21, 22] and *RAC2* [23, 24] cause distinct forms of PID. These examples illustrate the presence of allelic series and add to the complexity of genotype-phenotype relationships, which complicates correct variant interpretation using NGS.

## Practical Guide for WES Analysis

The utilization of WES in the diagnosis of PIDs has three clear advantages over targeted gene panels. Firstly, *in silico* gene panels used with WES can be adjusted as more PID-associated genes are identified, allowing for the analysis of new patients with the most up-to-date gene panel, and regular reanalysis of previously unsolved PID cases [9]. Filtering of the WES data provides a more straightforward analysis of known disease genes. Importantly, WES also facilitates the analysis of genes not included in any gene panel, providing the possibility to find candidate variants in all known coding regions of the genome in a research setting [2]. With this analysis, it is possible to identify rare variants in genes that could be involved in the phenotype of the patient in genes not yet associated with any form of PID. Secondly, WES provides genome wide detection of copy number variation (CNVs) [25, 26] and regions of homozygosity (ROHs) [27]. Thirdly, potentially relevant but unsubstantiated findings from the analysis of a single gene, gene panel, or *in silico* WES analysis can be more reliably interpreted after full exome or genome analysis to find better matching candidate variants. When no other better suitable candidate variant is found, this may support the suspicion of pathogenicity. In contrast, gene panels allow deeper sequencing, as only a selection of genes are sequenced, possibly leading to more reliable variant calling, especially for the detection of mosaicism [28]. WES also has a higher chance of incidental findings, requiring more intensive counseling by clinical geneticists [29].

**Fig. 2:**
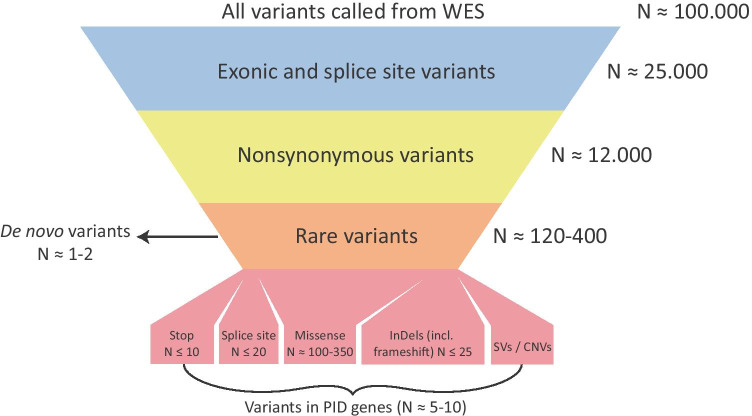
The general steps used to filter variants from WES. The approximate number of variants in each step is indicated. All filtering steps can be applied *in silico*. The number of variants that remain after filtering depends on the cut-off values used for filtering based on allele frequency and on algorithms used to call CNVs [3, 19, 26]. CNV numbers are highly dependent on the algorithm used; therefore, the number of CNVs is not indicated here. For the analysis of *de novo* variants, sequencing of a patient’s parents as a trio is required, after which all variants present in the parents can be filtered [3].

There is no single, most optimal approach to analyze exome data. Here, we aim to summarize the best practices from the literature and give practical guidance for this type of analysis. The analysis of variants detected using WES involves several filtering steps. Figure 2 shows these filtering steps schematically, with an approximate number of variants left after each step. These filter steps retain rare, coding non-synonymous variants, i.e. variants that alter the amino acid and variants affecting canonical splice sites that are rare in the general population. Rare variants are filtered based on variant allele frequencies (VAF), which are obtained from databases such as dbSNP and gnomAD [10, 11]. Also, variant frequencies from exomes in local and in-house databases can be useful to address local genomic variation or recurrent artifacts that may be platform specific [2, 30]. The exact allele frequency cut-off for filtering variants remains a matter of debate, but generally lies below 1%. Different frequencies may be applied for suspected dominant or recessive diseases [19]. A list of remaining variants can be ranked based on variant, gene, and available pedigree or segregation data, respectively. Variants are prioritized by the effect of the single-nucleotide variant (SNV) or CNV: frameshift, splice site, missense, insertion, and deletion. The remaining rare variants can be checked against a list of known PID genes and further analyzed based on predicted protein effect, conservation, constraint against LoF, and other annotations such as information of gene ontology (GO) and phenotypes of knockout mice models [31]. An approach that can significantly reduce the number of variants is the concurrent analysis of equally affected or unaffected close relatives. Especially the application of a trio WES analysis, which includes the healthy parents of the patient, can be especially relevant in (severe) sporadic cases of PID. *De novo* variants can be identified by excluding all variants inherited from either parent [3, 19]. Each exome harbors approximately 1–2 *de novo* variants, as indicated in Fig. 2 [14]. *De novo* variants can be included in the analysis when the pedigree suggests *de novo* occurrence. In consanguineous families, homozygosity mapping can be applied. This approach is based on the principle that pathogenic variants are relatively often present in homozygous regions formed from identity by descend, where both alleles share all variation [32].

## Yield of WES for PID Diagnostics

WES has in many cases replaced targeted gene panels and Sanger sequencing and forms a first-line diagnostic approach for PID patients. Although the knowledge base on PIDs continues to improve, the majority of PID patients do not receive a diagnosis using NGS approaches [2, 5]. In this section, we determine the current diagnostic yield of NGS for PID from the current literature and discuss approaches to improve the diagnostic yield. We collected studies that describe the application of WES, WGS, and other targeted NGS approaches in PID patient cohorts. The studies that were included are presented in Table 1.

**Table 1:** A summary of the studies included for the determination of the yield of NGS based approaches in the diagnosis of PIDs. To determine the diagnostic yield of WES when applied in PID patients, literature describing the application of WES, other targeted NGS approaches, and WGS for the diagnosis of cohorts of PID patients were collected. A recent review from Yska et al. described the application of NGS in the diagnosis of PIDs, where a diagnostic yield between 15 and 79% was reported based on 14 studies [5]. To estimate the diagnostic yield of WES in PIDs, PubMed and Embase were used to search for studies describing WES and other NGS approaches for PIDs. The keywords “PID” or “Primary immunodeficiency diseases” were used in combination with “WES,” “NGS,” or “WGS.” Studies describing cohorts of PID patients were included, while studies describing single patients or families were excluded. We expanded the selection from Yska et al. with 10 additional studies. Seven of the studies described the application of WES, two described the application of an NGS approach targeting known PID genes, and one described the application of WGS in the diagnosis of PID patients. From these 24 studies, the sequencing approach, number of genes sequenced using a targeted gene panel or included in the *in silico* analysis using WES, the number of included patients, and the number and percentage of patients diagnosed were extracted. The average yield of either NGS-based sequencing approaches or a WES-based approach is calculated from the yield of these studies. In two studies that employed WES, the whole exome was analyzed when there were no candidates found in a panel of genes associated with PIDs. The country where patient recruitment was done is also indicated.

							Yield (*N* (%))			
Authors	Year	Selection	Country	Approach	Genes (*N*)	N	Diagnostic	Whole exome	Total	Ref
Bisgin et al.	2018	PID	Turkey	Targeted gene panel	60	37	17 (46)		17 (46)	[47]
Erman et al.	2017	SCID	Turkey	Targeted gene panel	356	19	6 (33)		6 (33)	[38]
Rae et al.	2018	PID	UK	Targeted gene panel	242	27	13 (46)		13 (46)	[48]
Moens et al.	2014	Patients known disease-causing mutation or with agammaglobulinemia without BTK mutations	Sweden + Poland	Targeted gene panel	179	15	6 (40)		6 (40)	[49]
Stoddard et al.	2014	PID	USA	Targeted gene panel	173	120	18 (15)		18 (15)	[50]
Nijman et al.	2014	CID, ALPS, granulopenia, HLH/XLP	The Netherlands	Targeted gene panel	170	26	4 (15)		4 (15)	[51]
Al-Mousa et al.	2016	Suspected PID	Saudi Arabia	Targeted gene panel	162	139	35 (25)		35 (25)	[39]
Yu et al.	2016	SCID	USA	Targeted gene panel	46	20	14 (70)		14 (70)	[34]
Kojima et al.	2016	PID	Japan	Targeted gene panel	349	59	8 (14)		8 (14)	[52]
Gallo et al.	2016	Suspected PID	Italy	Targeted gene panel + WES	571	45	7 (16)		7 (16)	[41]
Abolhassani et al.	2018	CID	Iran	Targeted gene panel + WES	200	243	189 (79)		189 (79)	[33]
Suspitsin et al.	2020	Pediatric PID patients	Russia	Targeted gene panel	344	333	69 (21)		69 (21)	[53]
Batlle-Maso et al.	2020	Autoinflammatory diseases	Spain	WES	4813	22	5 (23)		5 (23)	[42]
Abolhassani et al.	2019	Primary antibody deficiency (CVID, agammaglobulinemia, HIGM, IGAD)	Iran	Targeted gene panel + WES	378	126	86 (68)		86 (68)	[37]
Arts et al.	2019	PID	The Netherlands, Saudi Arabia, Finland	WES	302	254	62 (24)	10 (4)	72 (28)	[2]
Simon et al.	2020	PID	Israel	WES	?	106	74 (70)		74 (70)	[36]
Stray-Pedersen et al.	2017	PID	USA + Norway	WES	475	278	110 (40)		110 (40)	[35]
Okano et al.	2020	PID patients with severe symptoms with negative previous genetic targeted screening	Japan	WES	430	136	36 (26.5)		36 (26.5)	[54]
Maffucci et al.	2016	CVID	USA	WES	269	50	15 (30)		15 (30)	[55]
Rudilla et al.	2019	PID	Spain	WES	260	61	12 (20)	7 (11)	19 (31)	[8]
Borghesi et al.	2020	Pediatric PID patients with sepsis	Switzerland	WES	240	176	35 (20)		35 (20)	[56]
DeValles-Ibanez et al.	2018	Pediatric CVID patients	Spain	WES	16	36	5–8 (15–24)		5–8 (15–24)	[57]
Mukda et al.	2017	HLH	Thailand	WES	12	25	12 (48)		12 (48)	[43]
Thaventhiran et al.	2020	PID	UK	WGS	NA	886	91 (10.3)		91 (10.3)	[18]

*ALPS* autoimmune lymphoproliferative syndrome, *BTK* Bruton’s tyrosine kinase, *(S)CID* (severe) combined immunodeficiency, *CVID* common variable immunodeficiency, *HIGM* hyper immunoglobulin M syndrome, *HLH* hemophagocytic lymphohistiocytosis, *IGAD* immunoglobulin A deficiency, *NA* not applicable, *PID* primary immunodeficiency, *WES* whole exome sequencing, *XLP* X-linked lymphoproliferative disease.

The average diagnostic yield of NGS was 29% (range 10–70%) and 38% specifically for WES (range 15–70%) in the context of PIDs (Table 1). The average yield indicates that in many cases, a majority of PID patients are not effectively diagnosed using NGS-based sequencing approaches such as WES.

The marked spread in diagnostic yield between studies can most likely be explained by variation in patient selection and is dependent on disease severity. Patient cohort characteristics differed widely between studies, with some using specific subsets of PID, whereas other studies describe a generic cohort of PID patients. Furthermore, the selection criteria for these patients were not always clearly described. The different patient populations influence the *a priori* chance of establishing a genetic diagnosis. For example, patients with a severe combined immunodeficiency (SCID) present early in life with severe immune defects and have an increased chance of a monogenic diagnosis. This is reflected in the studies that were used, with two of the studies with the highest yield (79 and 70%) describing a cohort of SCID patients [33, 34]. Moreover, one study describing a generic cohort of PID patients found a diagnostic rate of 100% for SCID patients [35]. Patients with a consanguineous background are more likely to carry homozygous variants, which can be more easily identified using WES. This bias is also observed in the studies included in this review, where some patient populations originated from countries with a high level of consanguinity, thereby influencing the chance of homozygous variants leading to PIDs [2, 33, 35–39]. In adults with milder symptoms, the likelihood for a true monogenic defect may be lower, which is reflected in a proportionally lower yield. The yield may be decreased due to the possibility of polygenic inheritance, acquired immunodeficiency, and environmental factors [40]. Patient selection also influences diagnostic yield because some forms of PID are more extensively described in literature, causing differences in the number of genes described per disease.

The sequencing approaches also varied between studies. Some studies used a targeted gene panel, while other studies applied WES. Several studies combined these approaches [33, 37, 41]. The number of genes that were analyzed varied from 46 to 356 for targeted gene panel approaches and between 12 and 4813 for WES-based approaches, with one study using all genes associated with human disease [42]. These numbers correlate with the specificity of the patient selection, where cohorts with SCID or HLH (hemophagocytic lymphohistiocytosis) patients require fewer genes to be analyzed, while still resulting in a relatively high diagnostic yield [34, 43]. The differences of analyzed genes between studies indicate the challenge of establishing universal gene panels for PIDs. Initiatives such PanelApp, GF-PID (Genetics First Primary Immunodeficiencies), and the IUIS that establish gene lists for PIDs attempt to unify this process for individual clinics [1, 5, 44]. Taken together, the described differences in patient cohort characteristics, sequencing approaches, and selected gene panels between studies do not permit to draw definitive conclusions about the current diagnostic yield of NGS in (subsets of) PIDs.

The characterization of novel PID genes in research setting and their implementation in the diagnostic PID panel have improved the diagnostic rate in recent years [1]. In two studies, a WES-based approach was used to identify variants in several genes not included in the IUIS classification, leading to an improvement of the yield with 4 and 11 percentage points, respectively [2, 8]. This approach could therefore lead to extra diagnoses in laboratories with the possibility to perform whole exome (re-)analysis in research setting.

Several intrinsic shortcomings of WES also influence the diagnostic yield. These include incomplete coverage of some genes [45], but more specifically also incomplete analysis of structural variants, CNVs, repeat expansions, and the lack of coverage of most non-coding regions. These shortcomings are caused by mapping errors, which are inherent to short read sequencing approaches when applied to repetitive regions and because of the lack of targeting of regions that are outside of the exons. Because these sources of genetic variation might play a role in human disease, there is a possibility that diagnoses in PIDs are missed based on WES data [25, 46]. Ultimately, it is expected in the future that WGS will offer a more complete test to assess both the coding and non-coding part of the genome, outweighing the extra costs posed by more complex analysis of the data and data storage.

There are various indications that non-coding regions of the genome may also play a role in the pathogenesis of PIDs. In a recent study by Thaventhiran et al., 1318 PID patients underwent WGS, in order to address the diagnostic challenges posed by PID patients presenting in adulthood with no apparent family history of the disease. With this approach, 91 patients (10.3%) with variants in coding regions of known PID genes were diagnosed. Moreover, an analysis of the non-coding genome identified deletions in regulatory elements, which were shown to contribute to the phenotype. Examples include a case where compound heterozygosity of *ARPC1B* was detected in a patient, caused by a coding nonsense variant and a deletion of a region containing the promoter. This study provides insight into the influence of multiple variants on the phenotype. In the cohort, 60 (6.8%) patients had a pathogenic *TNFRSF13B* (TACI) variant, with five also carrying a variant in another PID gene [18]. This study gives insight into the involvement of intergenic variation and the possibility of digenic and possibly polygenic causes of PIDs. As non-coding variants are not detected using a WES-based approach, this result also indicates the added value of a whole-genome approach, by improving diagnostic yield through establishing deleterious variants in the non-coding regions of the genome.

## Challenges in the Discovery of New PID Genes

Whole-exome sequencing permits the discovery of novel disease-causing variants, although establishing a causal relationship between variant and phenotype remains challenging [1, 3]. As sequencing technologies continue to improve with rapid and cost-effective sequencing of all variants in the exome, the rate limiting factor of research has become the interpretation of this great wealth of genomic information [58]. Variant and gene annotations, overlapping strategies, and functional validations are vital in this process. However, these annotations still require interpretation and do not always clearly indicate the structural and functional effects of variants. Moreover, the effects of splice site variants, insertions, deletions, and structural variants are more difficult to interpret than SNVs. Also, variants in genes with a completely unknown function or poorly described functions are difficult to link to a phenotype without functional studies [3].

The number of rare variants that are generated from the WES data is based on cut-off values for the frequencies of the variants in various databases that represent the general population. These values therefore influence what variants are considered in the analysis of an exome. A generalized workflow for this approach is shown in Fig. 2. These cut-off values are based on the expected occurrence of disease-causing variants in the general population. However, choosing the cut-off values in the analysis of WES data remains subjective and a matter of debate. Filtering out variants that are less rare could lead to missed diagnoses, as more common alleles could also cause disease in compound heterozygous state. An example of common variants that can cause a genetic disease is the ABCA4 variant p.Asn1868Ile which has an allele frequency of close to 7% in the European population and causes a monogenic form of blindness known as Stargardt disease in a compound heterozygous fashion [59]. Filtering variants using a database of exomes from the local population can be very effective to filter benign variants that might occur at a very low frequency in the world-wide population in international databases [30].

The selection of possible gene candidates is highly dependent on annotations, which causes a bias towards well-annotated genes and gene families with known functions. These annotations can include disease models such as gene knockouts in mice, which lead to potential for error because of the differences between model organisms and humans. The overlap in disease phenotypes in humans and model organisms is modeled in tools such as Exomiser, which uses this phenotype data to filter variants from exome data [60]. The description of the phenotype and the pedigree by the physician is of equal importance for a more accurate association of this phenotype with a gene functioning in a certain pathway. Additionally, a complete pedigree is important for predicting the mode of inheritance of the disease.

*In silico* predictions of variant effect tools such as PhyloP, CADD, and predictions of the constraint to LoF of genes such as LOEUF are often used to estimate the effect of variants and gene LoF on protein function and by extension the phenotype [11–13]. These factors indicate the conservation between species, the effect of missense variants on protein function, and an estimate of the tolerance of the gene and of specific residues to variants, respectively. These predictions should be used with caution, as variants that score highly using these metrics do not necessarily indicate a relation to the phenotype. On the contrary, variants with lower scores do not directly indicate the opposite. These predictions should be used as a guide in combination with functional annotations to estimate the relevance of variants. Instead of interpreting variants based on just one or a few important metrics, interpretation should be underpinned by a synthesis of (predicted) gene function from literature and other gene annotations, predictive metrics for the specific variant and the gene itself, the phenotype, and pedigree of the patient to gain insight into the effect of variants on the phenotype and to identify possible candidate variants.

An effective approach to decrease the number of candidate variants is trio analysis, which includes sequencing of the parents of a patient suspected a sporadic form of AD disease caused by a *de novo* mutation. As for other severe, sporadic diseases such as intellectual disability, patient-parent trios allow for the systematic detection of possible *de novo* mutations, which are not present in the healthy parents and arise during gametogenesis or early in embryogenesis [14, 15]. Other forms of segregation by WES data can also be helpful; however, correct phenotyping of the tested family members is of great importance, in order to select variants shared between family members with comparable phenotypes or to exclude shared variants from unaffected family members. Nevertheless, the benefit of a pedigree is influenced by incomplete penetrance, other genetic factors, and environmental factors, where a PID manifests only with exposure to a specific pathogen [40, 61].

Individual PIDs are rare, as some genetic defects have only been described in a single patient or a handful of patients in the literature [1]. This causes difficulties in the future diagnosis of PIDs, especially in patients with similar phenotypes, as the clinical presentation of PIDs can overlap significantly. Cohort-based studies of patients with similar phenotypes or with variants in the same gene can provide a more robust analysis of the underlying genetic causes. Notably, overlapping the variants of multiple patients with the same phenotype can also drastically reduce the number of candidate variants. This phenotype-first approach has been used in the past for the elucidation of genetic disorders, such as Miller syndrome, where WES was first applied to establish a pathogenic variant in a Mendelian disorder [62]. However, this approach is not feasible in patients with extremely rare or phenotypically heterogeneous disorders. WES enables a genetics-first approach for patient diagnosis. Data sharing platforms such as Genematcher from Matchmaker Exchange and initiatives such as Solve-RD (research project funded by the European Commission to solve the unsolved rare genetic diseases) and GF-PID can be helpful to identify patients that possibly share pathogenic variants in the same gene, increasing the chance of causality [2, 5, 16, 17]. Quality control of the analytic approach can also be accomplished through multi-center validation of variant interpretation [63]. Nevertheless, additional functional validation of the effect of a genetic variant on gene and protein level is paramount to establish a direct causal relationship and to give insight in the molecular mechanism of the disease. In PID patients, immune cells can easily be extracted from blood to be studied *ex vivo*, providing a non-invasive method for validation experiments. In case the function of the affected gene is unknown, its function and the effect of the variant can be studied in gene knockout and knock-in models to evaluate pathogenicity of either suspected LoF and hypermorphic or GoF variants, respectively.

WES can exclusively uncover the genetic causes of PIDs located in the coding regions of the genome. As described above, WGS allows for identification of variants in introns and regulatory elements and provides improved identification of structural variants such as copy number variants (CNVs) and other structural rearrangements [19, 64]. The high percentage of unsolved PID cases using WES, as determined from literature describing the application of WES for the diagnosis of PID patients, indicate there might be a possibility that the pathogenic variants are located in the non-coding regions of the genome. WGS has also been recently applied for the analysis of variants in PID patients. The added value of this approach was demonstrated, with the identification of compound heterozygous variants in both coding and non-coding regions [18]. However, the analysis of the vast amount of data generated by WGS is more complex than WES and more expensive, hindering its application in routine diagnostics [19]. Moreover, WGS has shown only a modest improvement of the diagnostic yield and coverage of coding sequences compared with WES so far [46]. WES and WGS both employ short read sequencing with GC bias leading to biased coverage. Short-read sequencing approaches suffer from mapping difficulties of repetitive and paralogous sequences. Long-read sequencing could be a potential solution, facilitating the sequencing of regions difficult to assess using short read NGS technologies [65].

**Fig. 3:**
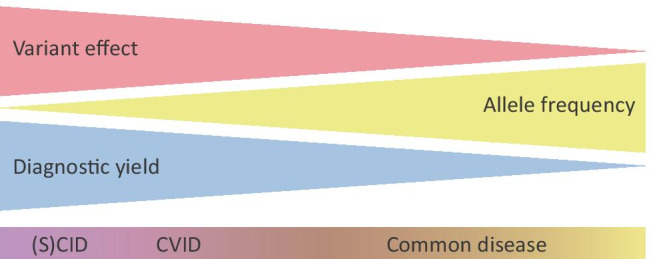
The relationship between variant effect size, allele frequency, and the diagnostic success rate in the field of PIDs. The triangles indicate variant effect, allele frequency, and diagnostic yield, ranging from highly impacting to weakly impacting, from extremely rare to common and from high to low, respectively. The characteristics of (severe) combined immunodeficiency ((S)CID), common variable immunodeficiency (CVID), and common disease are indicated in their approximate location within these three indicators

PIDs have long been considered as rare autosomal recessive disorders that cause completely penetrant phenotypes with severe defects in the immune system. However, this paradigm has shifted towards PIDs as a continuum ranging from mild and common diseases to severe and rare immune defects. This is shown in Fig. 3, which depicts the relationships between allele frequency of pathogenic variants, disease severity, and diagnostic yield in the context of PIDs. This indicates the spectrum formed by various forms of PID, ranging from extremely rare variants leading to severe disease with a high diagnostic yield ((S)CID), rare variants leading to a milder phenotype with an intermediate diagnostic yield (CVID), and more common variants that lead to common disease with a mild phenotype and a low diagnostic yield [61, 66]. The differences in the presentation of PIDs could be caused by the interplay of multiple variants in patients with milder symptoms, illustrated recently by the presence of TNFRSF13B (TACI) variants in PID patients that also presented with another pathogenic variant [18]. We postulate that genetic causes of PIDs are more common than previously thought and are inherited following more complex patterns. This constitutes a shift of PIDs from extremely rare recessive monogenic disorders that present in childhood towards a spectrum that includes more common diseases that are also caused by AD, *de novo,* multigenic and complex genetic variation that is also found in the non-coding regions of the genome and which causes disease later in life [40, 66, 67]. These factors also explain the limited diagnostic yield from NGS-based sequencing approaches, including WES, and indicate that this yield could be improved with more knowledge of these complex modes of inheritance that are being explored in the field of PIDs.

## Discussion

NGS-based sequencing approaches such as WES have been instrumental in the diagnosis of monogenic diseases such as PIDs, giving an insight into the underlying molecular disease mechanisms of immune defects [1, 5, 19, 37]. The heterogeneous nature of phenotypes seen in PID patients, combined with the influence of exposure to external factors, complicates the genetic diagnosis of these patients. Currently, more than 400 genes have been linked to inborn errors of immunity [1], with both clinical and genetic heterogeneity. Exome sequencing allows for the sequencing of the genomic regions that directly influence protein structure, which are highly relevant to human disease [3, 19]. Because of the efficient nature of NGS approaches, the interpretation of variants and their relation to patient phenotypes has become the rate limiting step in its application in clinical and research approaches [58]. We have evaluated the application of NGS as a diagnostic approach in PIDs by discussing the current yield, inherent shortcomings of WES, strategies and difficulties of variant analysis, and the promises of exploring the non-coding genome using WGS. Furthermore, strategies for improvement of the diagnostic yield were considered. The general considerations of the application of NGS for the diagnosis of PIDs and a guide for variant analysis using WES can be found in Box 1.

We have collected studies describing the application of WES and other targeted NGS approaches for the diagnosis of PID in patient cohorts. These approaches resulted in a diagnosis in 10–79% of cases with an average of 29% and 38% for WES alone. The high variability in diagnostic yield between studies and the low average yield of WES and other NGS approaches is caused by several factors, the most important being the widely varying patient cohort characteristics. Apart from the apparent methodological differences between the studies, the average low yield is also in part explained by the limitations of WES as well as a knowledge gap in the interpretation of the genes associated with PIDs. It is challenging to tie a phenotype to a specific variant, for example, due to uncertain variant effects, complex mutational mechanisms, digenic or polygenic inheritance, and unknown gene functions. Furthermore, causative variants that are not efficiently identified by WES, such as structural and non-coding variants, could be present in WES-negative PID patients, which were described in a recent study where WGS was applied in the diagnosis of PID patients, indicating the added value of WGS in this context [18]. Additionally, there could also be a role for more common variants, possibly interacting with rare variants, in the pathogenesis of PIDs. Future research should focus on the elucidation of these relatively unexplored mutational mechanisms that play a role in PIDs [65, 68]. The contribution of external factors on the presentation of PIDs, such as the interplay with specific pathogens at various stages of life, also requires further investigation [40]. Lastly, we expect more effort towards functional characterization of the molecular consequences of genetic variation to guide therapeutic approaches.

WES has contributed to a more accurate diagnosis of PID patients in the clinic, leading the transition towards personalized medicine. A genetic diagnosis of PID patients helps to end “diagnostic odysseys,” enables genetic counseling for family members, and can inform on treatment approaches [2]. Since the advent of NGS, and WES specifically, it has become clear that PIDs are moving away from rare, monogenic diseases with a severe clinical presentation often in childhood. As postulated by Casanova and Abel, PIDs might be more common than originally thought, with most individuals suffering from recurrent infections having some form of PID, with its presentation depending on the environment [61, 66]. The number of genes, genetic mechanisms, and phenotypes associated with PIDs keeps growing. This is in part due to the rise of novel pathogens, as exemplified by the COVID-19 pandemic during which pathogenic variants in both novel and known PID genes involved in the host immune response to viral infections have been associated with more severe forms of infection caused by the SARS-CoV-2 virus [69, 70]. PIDs give a unique insight into the molecular mechanisms of the immune response, shedding light on the pathways involved in the functioning and balance of immunity and self-tolerance. The continuing development in sequencing strategies and data interpretation will continue to improve the diagnosis and treatment of PID patients in the future.

## References

[1] Tangye SG, Al-Herz W, Bousfiha A, Chatila T, Cunningham-Rundles C, Etzioni A, Franco JL, Holland SM, Klein C, Morio T, Ochs HD, Oksenhendler E, Picard C, Puck J, Torgerson TR, Casanova JL, Sullivan KE (2020). Human inborn errors of immunity: 2019 update on the Classification from the International Union of Immunological Societies Expert Committee. J Clin Immunol.

[2] Arts P, Simons A, Alzahrani MS, Yilmaz E, Alidrissi E, Van Aerde KJ, Alenezi N, Alghamdi HA, Aljubab HA, Al-Hussaini AA, Almanjomi F, Alsaad AB, Alsaleem B, Andijani AA, Asery A, Ballourah W, Bleeker-Rovers CP, Van Deuren M, Van Der Flier M, Gerkes EH, Gilissen C, Habazi MK, Hehir-Kwa JY, Henriet SS, Hoppenreijs EP, Hortillosa S, Kerkhofs CH, Keski-Filppula R, Lelieveld SH, Lone K, MacKenzie MA, Mensenkamp AR, Moilanen J, Nelen M, Ten Oever J, Potjewijd J, Van Paassen P, Schuurs-Hoeijmakers JHM, Simon A, Stokowy T, Van De Vorst M, Vreeburg M, Wagner A, Van Well GTJ, Zafeiropoulou D, Zonneveld-Huijssoon E, Veltman JA, Van Zelst-Stams WAG, Faqeih EA, Van De Veerdonk FL, Netea MG, Hoischen A (2019). Exome sequencing in routine diagnostics: a generic test for 254 patients with primary immunodeficiencies. Genome Med.

[3] Gilissen C, Hoischen A, Brunner HG, Veltman JA (2012). Disease gene identification strategies for exome sequencing. Eur J Hum Genet.

[4] Ameratunga R, Lehnert K, Woon ST, Gillis D, Bryant VL, Slade CA, Steele R (2018). Review: diagnosing common variable immunodeficiency disorder in the era of genome sequencing. Clin Rev Allergy Immunol.

[5] Yska HAF, Elsink K, Kuijpers TW, Frederix GWJ, van Gijn ME, van Montfrans JM (2019). Diagnostic yield of next generation sequencing in genetically undiagnosed patients with primary immunodeficiencies: a systematic review. J Clin Immunol.

[6] Seleman M, Hoyos-Bachiloglu R, Geha RS, Chou J (2017). Uses of next-generation sequencing technologies for the diagnosis of primary immunodeficiencies. Front Immunol.

[7] Rabbani B, Tekin M, Mahdieh N (2014). The promise of whole-exome sequencing in medical genetics. J Hum Genet.

[8] Rudilla F, Franco-Jarava C, Martínez-Gallo M, Garcia-Prat M, Martín-Nalda A, Rivière J, Aguiló-Cucurull A, Mongay L, Vidal F, Solanich X, Irastorza I, Santos-Pérez JL, Tercedor Sánchez J, Cuscó I, Serra C, Baz-Redón N, Fernández-Cancio M, Carreras C, Vagace JM, Garcia-Patos V, Pujol-Borrell R, Soler-Palacín P, Colobran R (2019) Expanding the clinical and genetic spectra of primary immunodeficiency-related disorders with clinical exome sequencing: expected and unexpected findings. Front Immunol 10. 10.3389/fimmu.2019.02325 10.3389/fimmu.2019.02325 PMC6797824 31681265

[9] Wright CF, McRae JF, Clayton S, Gallone G, Aitken S, FitzGerald TW, Jones P, Prigmore E, Rajan D, Lord J, Sifrim A, Kelsell R, Parker MJ, Barrett JC, Hurles ME, FitzPatrick DR, Firth HV, Study DDD (2018). Making new genetic diagnoses with old data: iterative reanalysis and reporting from genome-wide data in 1,133 families with developmental disorders. Genet Med.

[10] Sherry ST, Ward M-H, Kholodov M, Baker J, Phan L, Smigielski EM, Sirotkin K (2001) dbSNP: the NCBI database of genetic variation. Nucleic Acids Res 29 (1):308-311. 10.1093/nar/29.1.308 10.1093/nar/29.1.308 PMC29783 11125122

[11] Karczewski KJ, Francioli LC, Tiao G, Cummings BB, Alfoldi J, Wang Q, Collins RL, Laricchia KM, Ganna A, Birnbaum DP, Gauthier LD, Brand H, Solomonson M, Watts NA, Rhodes D, Singer-Berk M, England EM, Seaby EG, Kosmicki JA, Walters RK, Tashman K, Farjoun Y, Banks E, Poterba T, Wang A, Seed C, Whiffin N, Chong JX, Samocha KE, Pierce-Hoffman E, Zappala Z, O'Donnell-Luria AH, Minikel EV, Weisburd B, Lek M, Ware JS, Vittal C, Armean IM, Bergelson L, Cibulskis K, Connolly KM, Covarrubias M, Donnelly S, Ferriera S, Gabriel S, Gentry J, Gupta N, Jeandet T, Kaplan D, Llanwarne C, Munshi R, Novod S, Petrillo N, Roazen D, Ruano-Rubio V, Saltzman A, Schleicher M, Soto J, Tibbetts K, Tolonen C, Wade G, Talkowski ME, Genome Aggregation Database C, Neale BM, Daly MJ, MacArthur DG (2020). The mutational constraint spectrum quantified from variation in 141,456 humans. Nature.

[12] Rentzsch P, Witten D, Cooper GM, Shendure J, Kircher M (2019). CADD: predicting the deleteriousness of variants throughout the human genome. Nucleic Acids Res.

[13] Pollard KS, Hubisz MJ, Rosenbloom KR, Siepel A (2010). Detection of nonneutral substitution rates on mammalian phylogenies. Genome Res.

[14] Veltman JA, Brunner HG (2012). De novo mutations in human genetic disease. Nat Rev Genet.

[15] Acuna-Hidalgo R, Veltman JA, Hoischen A (2016). New insights into the generation and role of de novo mutations in health and disease. Genome Biol.

[16] Buske OJ, Schiettecatte F, Hutton B, Dumitriu S, Misyura A, Huang L, Hartley T, Girdea M, Sobreira N, Mungall C, Brudno M (2015). The Matchmaker Exchange API: automating patient matching through the exchange of structured phenotypic and genotypic profiles. Hum Mutat.

[17] Sobreira N, Schiettecatte F, Valle D, Hamosh A (2015). GeneMatcher: a matching tool for connecting investigators with an interest in the same gene. Hum Mutat.

[18] Thaventhiran JED, Lango Allen H, Burren OS, Rae W, Greene D, Staples E, Zhang Z, Farmery JHR, Simeoni I, Rivers E, Maimaris J, Penkett CJ, Stephens J, Deevi SVV, Sanchis-Juan A, Gleadall NS, Thomas MJ, Sargur RB, Gordins P, Baxendale HE, Brown M, Tuijnenburg P, Worth A, Hanson S, Linger RJ, Buckland MS, Rayner-Matthews PJ, Gilmour KC, Samarghitean C, Seneviratne SL, Sansom DM, Lynch AG, Megy K, Ellinghaus E, Ellinghaus D, Jorgensen SF, Karlsen TH, Stirrups KE, Cutler AJ, Kumararatne DS, Chandra A, Edgar JDM, Herwadkar A, Cooper N, Grigoriadou S, Huissoon AP, Goddard S, Jolles S, Schuetz C, Boschann F, Abbs S, Adhya Z, Adlard J, Afzal M, Ahmed I, Ahmed M, Ahmed S, Aitman TJ, Alachkar H, Alamelu J, Alikhan R, Allen CE, Allen L, Allsup DJ, Alvi A, Ambegaonkar G, Anantharachagan A, Ancliff P, Anderson J, Antrobus R, Armstrong R, Arno G, Arumugakani G, Arya R, Ashford S, Astle WJ, Attwood A, Austin S, Aydinok Y, Ayub W, Babbs C, Bacchelli C, Baglin T, Bakchoul T, Bariana TK, Barratt J, Barwell J, Baski J, Bates RW, Batista J, Baxendale HE, Baynam G, Bennett DL, Bethune C, Bhatnagar N, Bibi S, Bierzynska A, Biss T, Bitner-Glindzicz MAK, Bleda M, Blesneac I, Boardman B, Boddana P, Bogaard HJ, Booth C, Boyce S, Bradley JR, Brady A, Breen G, Brennan P, Brewer C, Briley A, Brown M, Brown R, Browning MJ, Brownlie M, Bryson CJ, Buchan RJ, Buck J, Buckland MS, Bueser T, Diz CB, Burns SO, Burren OS, Calleja P, Carmichael J, Carr-White G, Carss KJ, Casey R, Chalmers E, Chambers J, Chambers J, Chan MMY, Chan MV, Chandra A, Cheng F, Chinn IK, Chinnery PF, Chitre M, Chong S, Christian MT, Church C, Clement EM, Brod NC, Clifford H, Clowes VE, Coghlan G, Colby E, Cole TRP, Collins JH, Collins PW, Condliffe R, Cook HT, Cook S, Cookson V, Cooper N, Corris PA, Creaser-Myers A, Crisp-Hihn A, Curry NS, Cutler AJ, Da Costa R, Danesino C, Daniels MJ, Darby D, Daugherty LC, Davies EG, Davies S, Davis J, de Bree GJ, Deacock S, Deegan PB, Deevi SVV, Dempster J, Dent T, Deshpande C, Devlin LA, Dewhurst EF, Dixit AK, Dixon PH, Doffinger R, Dolling H, Dormand N, Downes K, Drazyk AM, Drewe E, Duarte D, Dutt T, Edgar JDM, Edwards KE, Egner W, Ekani MN, El-Shanawany T, Elkhalifa S, Elston T, Emmerson I, Erber WN, Erwood M, Estiu MC, Evans DG, Evans G, Everington T, Eyries M, Farmery JHR, Favier R, Firth HV, Fitzpatrick MM, Fletcher D, Flinter FA, Fox JC, Frary AJ, French CE, Freson K, Frontini M, Furie B, Gale DP, Gall HJ, Gardham A, Gaspar HB, Gattens M, Ghali N, Ghataorhe PK, Ghio S, Ghofrani H-A, Ghurye R, Gibbs JSR, Gilbert RD, Gilmour KC, Girerd B, Girling JC, Gissen P, Gleadall NS, Goddard S, Gordins P, Gorman KM, Gosal D, Graf S, Grassi L, Greene D, Greenhalgh AJ, Greenhalgh L, Greinacher A, Gresele P, Griffiths PG, Griffiths S, Grigoriadou S, Grozeva D, Hackett SJ, Hadden RDM, Hadinnapola C, Hague R, Hague WM, Haimel M, Hall M, Halmagyi C, Hammerton T, Hanson HL, Harkness K, Harper AR, Harper L, Harris C, Harrison C, Hart D, Hassan A, Hayman G, Heemskerk JWM, Hegde S, Henderson A, Henderson RH, Hensiek A, Henskens YMC, Herwadkar A, Hodgson J, Hoffman J, Holden S, Holder M, Horvath R, Houlden H, Houweling AC, Howard LS, Hu F, Hudson G, Hughes S, Hughes S, Huis in ‘t Veld AE, Huissoon AP, Humbert M, Hurles ME, Hurst JA, Irvine V, Izatt L, James R, Jeevaratnam P, Johnson M, Johnson SA, Jolles S, Jolley JD, Jones B, Jones J, Bioresource PICftN,  (2020). Whole-genome sequencing of a sporadic primary immunodeficiency cohort. Nature.

[19] Meyts I, Bosch B, Bolze A, Boisson B, Itan Y, Belkadi A, Pedergnana V, Moens L, Picard C, Cobat A, Bossuyt X, Abel L, Casanova JL (2016). Exome and genome sequencing for inborn errors of immunity. J Allergy Clin Immunol.

[20] Lu HY, Bauman BM, Arjunaraja S, Dorjbal B, Milner JD, Snow AL, Turvey SE (2018) The CBM-opathies—a rapidly expanding spectrum of human inborn errors of immunity caused by mutations in the CARD11-BCL10-MALT1 complex. Front Immunol 9. 10.3389/fimmu.2018.02078 10.3389/fimmu.2018.02078 PMC6156466 30283440

[21] Toubiana J, Okada S, Hiller J, Oleastro M, Gomez ML, Becerra JCA, Ouachée-Chardin M, Fouyssac F, Girisha KM, Etzioni A, Van Montfrans J, Camcioglu Y, Kerns LA, Belohradsky B, Blanche S, Bousfiha A, Rodriguez-Gallego C, Meyts I, Kisand K, Reichenbach J, Renner ED, Rosenzweig S, Grimbacher B, Van De Veerdonk FL, Traidl-Hoffmann C, Picard C, Marodi L, Morio T, Kobayashi M, Lilic D, Milner JD, Holland S, Casanova JL, Puel A (2016). Heterozygous STAT1 gain-of-function mutations underlie an unexpectedly broad clinical phenotype. Blood.

[22] Van De Veerdonk FL, Plantinga TS, Hoischen A, Smeekens SP, Joosten LAB, Gilissen C, Arts P, Rosentul DC, Carmichael AJ, Smits-van Der Graaf CAA, Kullberg BJ, Van Der Meer JWM, Lilic D, Veltman JA, Netea MG (2011). STAT1 mutations in autosomal dominant chronic mucocutaneous candidiasis. N Engl J Med.

[23] Alkhairy OK, Rezaei N, Graham RR, Abolhassani H, Borte S, Hultenby K, Wu C, Aghamohammadi A, Williams DA, Behrens TW, Hammarström L, Pan-Hammarström Q (2015). RAC2 loss-of-function mutation in 2 siblings with characteristics of common variable immunodeficiency. J Allergy Clin Immunol.

[24] Smits BM, Lelieveld PHC, Ververs FA, Turkenburg M, de Koning C, van Dijk M, Leavis HL, Boelens JJ, Lindemans CA, Bloem AC, van de Corput L, van Montfrans J, Nierkens S, van Gijn ME, Geerke DP, Waterham HR, Koenderman L, Boes M (2020). A dominant activating RAC2 variant associated with immunodeficiency and pulmonary disease. Clinical Immunology.

[25] Gambin T, Akdemir ZC, Yuan B, Gu S, Chiang T, Carvalho CMB, Shaw C, Jhangiani S, Boone PM, Eldomery MK, Karaca E, Bayram Y, Stray-Pedersen A, Muzny D, Charng WL, Bahrambeigi V, Belmont JW, Boerwinkle E, Beaudet AL, Gibbs RA, Lupski JR (2017). Homozygous and hemizygous CNV detection from exome sequencing data in a Mendelian disease cohort. Nucleic Acids Res.

[26] Krumm N, Sudmant PH, Ko A, O'Roak BJ, Malig M, Coe BP, Project NES, Quinlan AR, Nickerson DA, Eichler EE (2012). Copy number variation detection and genotyping from exome sequence data. Genome Res.

[27] Pippucci T, Magi A, Gialluisi A, Romeo G (2014). Detection of runs of homozygosity from whole exome sequencing data: State of the art and perspectives for clinical, population and epidemiological studies. Hum Hered.

[28] LaDuca H, Farwell KD, Vuong H, Lu HM, Mu W, Shahmirzadi L, Tang S, Chen J, Bhide S, Chao EC (2017). Exome sequencing covers >98% of mutations identified on targeted next generation sequencing panels. PLoS One.

[29] Kalia SS, Adelman K, Bale SJ, Chung WK, Eng C, Evans JP, Herman GE, Hufnagel SB, Klein TE, Korf BR, McKelvey KD, Ormond KE, Richards CS, Vlangos CN, Watson M, Martin CL, Miller DT (2017) Recommendations for reporting of secondary findings in clinical exome and genome sequencing, 2016 update (ACMG SF v2.0): a policy statement of the American College of Medical Genetics and Genomics. Genet Med 19 (2):249–255. 10.1038/gim.2016.190 10.1038/gim.2016.190 27854360

[30] Francioli LC, Menelaou A, Pulit SL, van Dijk F, Palamara PF, Elbers CC, Neerincx PBT, Ye K, Guryev V, Kloosterman WP, Deelen P, Abdellaoui A, van Leeuwen EM, van Oven M, Vermaat M, Li M, Laros JFJ, Karssen LC, Kanterakis A, Amin N, Hottenga JJ, Lameijer E-W, Kattenberg M, Dijkstra M, Byelas H, van Setten J, van Schaik BDC, Bot J, Nijman IJ, Renkens I, Marschall T, Schönhuth A, Hehir-Kwa JY, Handsaker RE, Polak P, Sohail M, Vuzman D, Hormozdiari F, van Enckevort D, Mei H, Koval V, Moed MH, van der Velde KJ, Rivadeneira F, Estrada K, Medina-Gomez C, Isaacs A, McCarroll SA, Beekman M, de Craen AJM, Suchiman HED, Hofman A, Oostra B, Uitterlinden AG, Willemsen G, Study LC, Platteel M, Veldink JH, van den Berg LH, Pitts SJ, Potluri S, Sundar P, Cox DR, Sunyaev SR, Dunnen JTd, Stoneking M, de Knijff P, Kayser M, Li Q, Li Y, Du Y, Chen R, Cao H, Li N, Cao S, Wang J, Bovenberg JA, Pe'er I, Slagboom PE, van Duijn CM, Boomsma DI, van Ommen G-JB, de Bakker PIW, Swertz MA, Wijmenga C, The Genome of the Netherlands C 2014 Whole-genome sequence variation, population structure and demographic history of the Dutch population Nat Genet 46 8 818 825. 10.1038/ng.3021

[31] The Gene Ontology Consortium (2018). The gene ontology resource: 20 years and still GOing strong. Nucleic Acids Res.

[32] Wakeling MN, Laver TW, Wright CF, De Franco E, Stals KL, Patch AM, Hattersley AT, Flanagan SE, Ellard S, Study DDD (2019). Homozygosity mapping provides supporting evidence of pathogenicity in recessive Mendelian disease. Genet Med.

[33] Abolhassani H, Chou J, Bainter W, Platt CD, Tavassoli M, Momen T, Tavakol M, Eslamian MH, Gharagozlou M, Movahedi M, Ghadami M, Hamidieh AA, Azizi G, Yazdani R, Afarideh M, Ghajar A, Havaei A, Chavoshzadeh Z, Mahdaviani SA, Cheraghi T, Behniafard N, Amin R, Aleyasin S, Faridhosseini R, Jabbari-Azad F, Nabavi M, Bemanian MH, Arshi S, Molatefi R, Sherkat R, Mansouri M, Mesdaghi M, Babaie D, Mohammadzadeh I, Ghaffari J, Shafiei A, Kalantari N, Ahanchian H, Khoshkhui M, Soheili H, Dabbaghzadeh A, Shirkani A, Nasiri Kalmarzi R, Mortazavi SH, Tafaroji J, Khalili A, Mohammadi J, Negahdari B, Joghataei MT, al-Ramadi BK, Picard C, Parvaneh N, Rezaei N, Chatila TA, Massaad MJ, Keles S, Hammarström L, Geha RS, Aghamohammadi A,  (2018). Clinical, immunologic, and genetic spectrum of 696 patients with combined immunodeficiency. J Allergy Clin Immunol.

[34] Yu H, Zhang VW, Stray-Pedersen A, Hanson IC, Forbes LR, de la Morena MT, Chinn IK, Gorman E, Mendelsohn NJ, Pozos T, Wiszniewski W, Nicholas SK, Yates AB, Moore LE, Berge KE, Sorte H, Bayer DK, ALZahrani D, Geha RS, Feng Y, Wang G, Orange JS, Lupski JR, Wang J, Wong LJ,  (2016). Rapid molecular diagnostics of severe primary immunodeficiency determined by using targeted next-generation sequencing. J Allergy Clin Immunol.

[35] Stray-Pedersen A, Sorte HS, Samarakoon P, Gambin T, Chinn IK, Coban Akdemir ZH, Erichsen HC, Forbes LR, Gu S, Yuan B, Jhangiani SN, Muzny DM, Rødningen OK, Sheng Y, Nicholas SK, Noroski LM, Seeborg FO, Davis CM, Canter DL, Mace EM, Vece TJ, Allen CE, Abhyankar HA, Boone PM, Beck CR, Wiszniewski W, Fevang B, Aukrust P, Tjønnfjord GE, Gedde-Dahl T, Hjorth-Hansen H, Dybedal I, Nordøy I, Jørgensen SF, Abrahamsen TG, Øverland T, Bechensteen AG, Skogen V, Osnes LTN, Kulseth MA, Prescott TE, Rustad CF, Heimdal KR, Belmont JW, Rider NL, Chinen J, Cao TN, Smith EA, Caldirola MS, Bezrodnik L, Lugo Reyes SO, Espinosa Rosales FJ, Guerrero-Cursaru ND, Pedroza LA, Poli CM, Franco JL, Trujillo Vargas CM, Aldave Becerra JC, Wright N, Issekutz TB, Issekutz AC, Abbott J, Caldwell JW, Bayer DK, Chan AY, Aiuti A, Cancrini C, Holmberg E, West C, Burstedt M, Karaca E, Yesil G, Artac H, Bayram Y, Atik MM, Eldomery MK, Ehlayel MS, Jolles S, Flatø B, Bertuch AA, Hanson IC, Zhang VW, Wong LJ, Hu J, Walkiewicz M, Yang Y, Eng CM, Boerwinkle E, Gibbs RA, Shearer WT, Lyle R, Orange JS, Lupski JR (2017). Primary immunodeficiency diseases: genomic approaches delineate heterogeneous Mendelian disorders. J Allergy Clin Immunol.

[36] Simon AJ, Golan AC, Lev A, Stauber T, Barel O, Somekh I, Klein C, AbuZaitun O, Eyal E, Kol N, Unal E, Amariglio N, Rechavi G, Somech R (2020). Whole exome sequencing (WES) approach for diagnosing primary immunodeficiencies (PIDs) in a highly consanguineous community. Clinical Immunology.

[37] Abolhassani H, Aghamohammadi A, Fang M, Rezaei N, Jiang C, Liu X, Pan-Hammarström Q, Hammarström L (2019). Clinical implications of systematic phenotyping and exome sequencing in patients with primary antibody deficiency. Genet Med.

[38] Erman B, Bilic I, Hirschmugl T, Salzer E, Boztug H, Sanal ÇA, D, Tezcan I, Boztug K,  (2017). Investigation of genetic defects in severe combined immunodeficiency patients from turkey by targeted sequencing. Scand J Immunol.

[39] Al-Mousa H, Abouelhoda M, Monies DM, Al-Tassan N, Al-Ghonaium A, Al-Saud B, Al-Dhekri H, Arnaout R, Al-Muhsen S, Ades N, Elshorbagi S, Al Gazlan S, Sheikh F, Dasouki M, El-Baik L, Elamin T, Jaber A, Kheir O, El-Kalioby M, Subhani S, Al Idrissi E, Al-Zahrani M, Alhelale M, Alnader N, Al-Otaibi A, Kattan R, Al Abdelrahman K, Al Breacan MM, Bin Humaid FS, Wakil SM, Alzayer F, Al-Dusery H, Faquih T, Al-Hissi S, Meyer BF, Hawwari A (2016). Unbiased targeted next-generation sequencing molecular approach for primary immunodeficiency diseases. J Allergy Clin Immunol.

[40] Casanova JL (2015). Severe infectious diseases of childhood as monogenic inborn errors of immunity. Proc Natl Acad Sci U S A.

[41] Gallo V, Dotta L, Giardino G, Cirillo E, Lougaris V, D'Assante R, Prandini A, Consolini R, Farrow EG, Thiffault I, Saunders CJ, Leonardi A, Plebani A, Badolato R, Pignata C (2016). Diagnostics of primary immunodeficiencies through next-generation sequencing. Front Immunol.

[42] Batlle-Masó L, Mensa-Vilaró A, Solís-Moruno M, Marquès-Bonet T, Arostegui JI, Casals F (2020). Genetic diagnosis of autoinflammatory disease patients using clinical exome sequencing. Eur J Med Genet.

[43] Mukda E, Trachoo O, Pasomsub E, Tiyasirichokchai R, Iemwimangsa N, Sosothikul D, Chantratita W, Pakakasama S (2017). Exome sequencing for simultaneous mutation screening in children with hemophagocytic lymphohistiocytosis. Int J Hematol.

[44] Martin AR, Williams E, Foulger RE, Leigh S, Daugherty LC, Niblock O, Leong IUS, Smith KR, Gerasimenko O, Haraldsdottir E, Thomas E, Scott RH, Baple E, Tucci A, Brittain H, de Burca A, Ibanez K, Kasperaviciute D, Smedley D, Caulfield M, Rendon A, McDonagh EM (2019). PanelApp crowdsources expert knowledge to establish consensus diagnostic gene panels. Nat Genet.

[45] Ebbert MTW, Jensen TD, Jansen-West K, Sens JP, Reddy JS, Ridge PG, Kauwe JSK, Belzil V, Pregent L, Carrasquillo MM, Keene D, Larson E, Crane P, Asmann YW, Ertekin-Taner N, Younkin SG, Ross OA, Rademakers R, Petrucelli L, Fryer JD (2019). Systematic analysis of dark and camouflaged genes reveals disease-relevant genes hiding in plain sight. Genome Biol.

[46] Barbitoff YA, Polev DE, Glotov AS, Serebryakova EA, Shcherbakova IV, Kiselev AM, Kostareva AA, Glotov OS, Predeus AV (2020). Systematic dissection of biases in whole-exome and whole-genome sequencing reveals major determinants of coding sequence coverage. Sci Rep.

[47] Bisgin A, Boga I, Yilmaz M, Bingol G, Altintas D (2018) The Utility of Next-Generation Sequencing for Primary Immunodeficiency Disorders: Experience from a Clinical Diagnostic Laboratory. Biomed Res Int. 10.1155/2018/9647253 10.1155/2018/9647253 PMC5977064 29888287

[48] Rae W, Ward D, Mattocks C, Pengelly RJ, Eren E, Patel SV, Faust SN, Hunt D, Williams AP (2018). Clinical efficacy of a next-generation sequencing gene panel for primary immunodeficiency diagnostics. Clin Genet.

[49] Moens LN, Falk-Sörqvist E, Asplund AC, Bernatowska E, Smith CIE, Nilsson M (2014). Diagnostics of primary immunodeficiency diseases: a sequencing capture approach. PLoS One.

[50] Stoddard JL, Niemela JE, Fleisher TA, Rosenzweig SD (2014). Targeted NGS: a cost-effective approach to molecular diagnosis of PIDs. Front Immunol.

[51] Nijman IJ, Van Montfrans JM, Hoogstraat M, Boes ML, Van De Corput L, Renner ED, Van Zon P, Van Lieshout S, Elferink MG, Van Der Burg M, Vermont CL, Van Der Zwaag B, Janson E, Cuppen E, Ploos Van Amstel JK, Van Gijn ME (2014). Targeted next-generation sequencing: A novel diagnostic tool for primary immunodeficiencies. J Allergy Clin Immunol.

[52] Kojima D, Wang X, Muramatsu H, Okuno Y, Nishio N, Hama A, Tsuge I, Takahashi Y, Kojima S (2016) Application of extensively targeted next-generation sequencing for the diagnosis of primary immunodeficiencies. J Allergy Clin Immunol 138 (1):303–305 e303. 10.1016/j.jaci.2016.01.012 10.1016/j.jaci.2016.01.012 26997321

[53] Suspitsin EN, Guseva MN, Kostik MM, Sokolenko AP, Skripchenko NV, Levina AS, Goleva OV, Dubko MF, Tumakova AV, Makhova MA, Lyazina LV, Bizin IV, Sokolova NE, Gabrusskaya TV, Ditkovskaya LV, Kozlova OP, Vahliarskaya SS, Kondratenko IV, Imyanitov EN (2020) Next generation sequencing analysis of consecutive Russian patients with clinical suspicion of inborn errors of immunity. Clin Genet:1–9.10.1111/cge.13789 10.1111/cge.13789 32441320

[54] Okano T, Imai K, Naruto T, Okada S, Yamashita M, Tw Y, Ono S, Tanaka K, Okamoto K, Tanita K, Matsumoto K, Toyofuku E, Kumaki-Matsumoto E, Okamura M, Ueno H, Ogawa S, Ohara O, Takagi M, Kanegane H, Morio T (2020). Whole-exome sequencing-based approach for germline mutations in patients with inborn errors of immunity. J Clin Immunol.

[55] Maffucci P, Filion CA, Boisson B, Itan Y, Shang L, Casanova JL, Cunningham-Rundles C (2016) Genetic diagnosis using whole exomesequencing in common variable immunodeficiency. Front Immunol 7. 10.3389/fimmu.2016.00220 10.3389/fimmu.2016.00220 PMC4903998 27379089

[56] Borghesi A, Trück J, Asgari S, Sancho-Shimizu V, Agyeman PKA, Bellos E, Giannoni E, Stocker M, Posfay-Barbe KM, Heininger U, Bernhard-Stirnemann S, Niederer-Loher A, Kahlert CR, Natalucci G, Relly C, Riedel T, Kuehni CE, Thorball CW, Chaturvedi N, Martinon-Torres F, Kuijpers TW, Coin L, Wright V, Herberg J, Levin M, Aebi C, Berger C, Fellay J, Schlapbach LJ (2020) Whole-exome Sequencing for the Identification of Rare Variants in Primary Immunodeficiency Genes in Children With Sepsis: A Prospective, Population-based Cohort Study. Clin Infect Dis:1–10. 10.1093/cid/ciaa290 10.1093/cid/ciaa290 PMC7744985 32185379

[57] de Valles-Ibáñez G, Esteve-Solé A, Piquer M, Azucena González-Navarro E, Hernandez-Rodriguez J, Laayouni H, González-Roca E, Plaza-Martin AM, Deyà-Martínez Á, Martín-Nalda A, Martínez-Gallo M, García-Prat M, del Pino-Molina L, Cuscó I, Codina-Solà M, Batlle-Masó L, Solís-Moruno M, Marquès-Bonet T, Bosch E, López-Granados E, Aróstegui JI, Soler-Palacín P, Colobran R, Yagüe J, Alsina L, Juan M, Casals F (2018) Evaluating the genetics of common variable immunodeficiency: monogenetic model and beyond. Front Immunol 9. 10.3389/fimmu.2018.00636 10.3389/fimmu.2018.00636 PMC5960686 29867916

[58] Cooper GM, Shendure J (2011). Needles in stacks of needles: finding disease-causal variants in a wealth of genomic data. Nat Rev Genet.

[59] Runhart EH, Sangermano R, Cornelis SS, Verheij J, Plomp AS, Boon CJF, Lugtenberg D, Roosing S, Bax NM, Blokland EAW, Jacobs-Camps MHM, van der Velde-Visser SD, Pott JR, Rohrschneider K, Thiadens A, Klaver CCW, van den Born LI, Hoyng CB, Cremers FPM (2018) The Common ABCA4 Variant p.Asn1868Ile Shows Nonpenetrance and Variable Expression of Stargardt Disease When Present in trans With Severe Variants. Invest Ophthalmol Vis Sci 59 (8):3220–3231.10.1167/iovs.18-23881 10.1167/iovs.18-23881 29971439

[60] Robinson PN, Kohler S, Oellrich A, Sanger Mouse Genetics P, Wang K, Mungall CJ, Lewis SE, Washington N, Bauer S, Seelow D, Krawitz P, Gilissen C, Haendel M, Smedley D (2014). Improved exome prioritization of disease genes through cross-species phenotype comparison. Genome Res.

[61] Casanova J-L, Abel L (2007) Primary Immunodeficiencies: A Field in Its Infancy. Science (80- ) 317:617 LP - 619. 10.1126/science.1142963 10.1126/science.1142963 17673650

[62] Ng SB, Buckingham KJ, Lee C, Bigham AW, Tabor HK, Dent KM, Huff CD, Shannon PT, Jabs EW, Nickerson DA, Shendure J, Bamshad MJ (2010). Exome sequencing identifies the cause of a mendelian disorder. Nat Genet.

[63] Elsink K, Huibers MMH, Hollink I, van der Veken LT, Ernst RF, Simons A, Zonneveld-Huijssoon E, van der Hout AH, Abbott KM, Hoischen A, Pieterse M, Kuijpers TW, van Montfrans JM, van Gijn ME (2020). National external quality assessment for next-generation sequencing-based diagnostics of primary immunodeficiencies. Eur J Hum Genet.

[64] Belkadi A, Bolze A, Itan Y, Cobat A, Vincent QB, Antipenko A, Shang L, Boisson B, Casanova JL, Abel L (2015). Whole-genome sequencing is more powerful than whole-exome sequencing for detecting exome variants. Proc Natl Acad Sci U S A.

[65] Mantere T, Kersten S, Hoischen A (2019). Long-Read Sequencing Emerging in Medical Genetics. Front Genet.

[66] van der Made CI, Hoischen A, Netea MG, van de Veerdonk FL (2020). Primary immunodeficiencies in cytosolic pattern-recognition receptor pathways: toward host-directed treatment strategies. Immunol Rev.

[67] Bousfiha AA, Jeddane L, Ailal F, Benhsaien I, Mahlaoui N, Casanova JL, Abel L (2013). Primary immunodeficiency diseases worldwide: more common than generally thought. J Clin Immunol.

[68] Revy P, Kannengiesser C, Fischer A (2019). Somatic genetic rescue in Mendelian haematopoietic diseases. Nat Rev Genet.

[69] van der Made CI, Simons A, Schuurs-Hoeijmakers J, van den Heuvel G, Mantere T, Kersten S, van Deuren RC, Steehouwer M, van Reijmersdal SV, Jaeger M, Hofste T, Astuti G, Corominas Galbany J, van der Schoot V, van der Hoeven H, Have HOT, W, Klijn E, van den Meer C, Fiddelaers J, de Mast Q, Bleeker-Rovers CP, Joosten LAB, Yntema HG, Gilissen C, Nelen M, van der Meer JWM, Brunner HG, Netea MG, van de Veerdonk FL, Hoischen A,  (2020). Presence of Genetic Variants Among Young Men With Severe COVID-19. JAMA.

[70] Zhang Q, Bastard P, Liu Z, Le Pen J, Moncada-Velez M, Chen J, Ogishi M, Sabli IKD, Hodeib S, Korol C, Rosain J, Bilguvar K, Ye J, Bolze A, Bigio B, Yang R, Arias AA, Zhou Q, Zhang Y, Onodi F, Korniotis S, Karpf L, Philippot Q, Chbihi M, Bonnet-Madin L, Dorgham K, Smith N, Schneider WM, Razooky BS, Hoffmann H-H, Michailidis E, Moens L, Han JE, Lorenzo L, Bizien L, Meade P, Neehus A-L, Ugurbil AC, Corneau A, Kerner G, Zhang P, Rapaport F, Seeleuthner Y, Manry J, Masson C, Schmitt Y, Schlüter A, Le Voyer T, Khan T, Li J, Fellay J, Roussel L, Shahrooei M, Alosaimi MF, Mansouri D, Al-Saud H, Al-Mulla F, Almourfi F, Al-Muhsen SZ, Alsohime F, Al Turki S, Hasanato R, van de Beek D, Biondi A, Bettini LR, D’Angio M, Bonfanti P, Imberti L, Sottini A, Paghera S, Quiros-Roldan E, Rossi C, Oler AJ, Tompkins MF, Alba C, Vandernoot I, Goffard J-C, Smits G, Migeotte I, Haerynck F, Soler-Palacin P, Martin-Nalda A, Colobran R, Morange P-E, Keles S, Çölkesen F, Ozcelik T, Yasar KK, Senoglu S, Karabela ŞN, Gallego CR, Novelli G, Hraiech S, Tandjaoui-Lambiotte Y, Duval X, Laouénan C, Snow AL, Dalgard CL, Milner J, Vinh DC, Mogensen TH, Marr N, Spaan AN, Boisson B, Boisson-Dupuis S, Bustamante J, Puel A, Ciancanelli M, Meyts I, Maniatis T, Soumelis V, Amara A, Nussenzweig M, García-Sastre A, Krammer F, Pujol A, Duffy D, Lifton R, Zhang S-Y, Gorochov G, Béziat V, Jouanguy E, Sancho-Shimizu V, Rice CM, Abel L, Notarangelo LD, Cobat A, Su HC, Casanova J-L (2020) Inborn errors of type I IFN immunity in patients with life-threatening COVID-19. Science (80- ):eabd4570. 10.1126/science.abd4570

